# Author Correction: Lipid exposure activates gene expression changes associated with estrogen receptor negative breast cancer

**DOI:** 10.1038/s41523-023-00516-3

**Published:** 2023-03-13

**Authors:** Shivangi Yadav, Ranya Virk, Carolina H. Chung, Mariana Bustamante Eduardo, David VanDerway, Duojiao Chen, Kirsten Burdett, Hongyu Gao, Zexian Zeng, Manish Ranjan, Gannon Cottone, Xiaoling Xuei, Sriram Chandrasekaran, Vadim Backman, Robert Chatterton, Seema Ahsan Khan, Susan E. Clare

**Affiliations:** 1 Department of Surgery, Feinberg School of Medicine, Northwestern University, Chicago, IL 60611 USA; 2 Department of Biomedical Engineering, Northwestern University, Evanston, IL 60208-2850 USA; 3 Department of Biomedical Engineering, University of Michigan, Ann Arbor, MI 48109 USA; 4 Center of for Medical Genomics, Indiana University School of Medicine, Indianapolis, IN 46202 USA; 5 Department of Preventive Medicine, Northwestern University, Chicago, IL 60611 USA; 6 Department of Data Sciences, Dana Farber Cancer Institute, Harvard T.H. Chan School of Public Health, Boston, MA 02215 USA; 7 Program in Chemical Biology, University of Michigan, Ann Arbor, MI 48109 USA; 8 Rogel Cancer Center, University of Michigan Medical School, Ann Arbor, MI 48109 USA; 9 Center for Computational Medicine and Bioinformatics, University of Michigan, Ann Arbor, MI 48109 USA; 10 Department of Obstetrics and Gynecology, Northwestern University Feinberg School of Medicine, Chicago, IL 60611 USA

**Keywords:** Breast cancer, Chromatin remodelling

Correction to: *npj Breast Cancer* 10.1038/s41523-022-00422-0, published online 04 May 2022

In this article, funding from the National Institutes of Health (award number R01CA228272) was inadvertently omitted. The original article has been corrected.

