# Supplementary material for: Lipid exposure activates gene expression changes associated with estrogen receptor negative breast cancer
Source: NPJ Breast Cancer. 2022 May 4;8:59. doi: 10.1038/s41523-022-00422-0 (PMC9068822; doi:10.1038/s41523-022-00422-0)

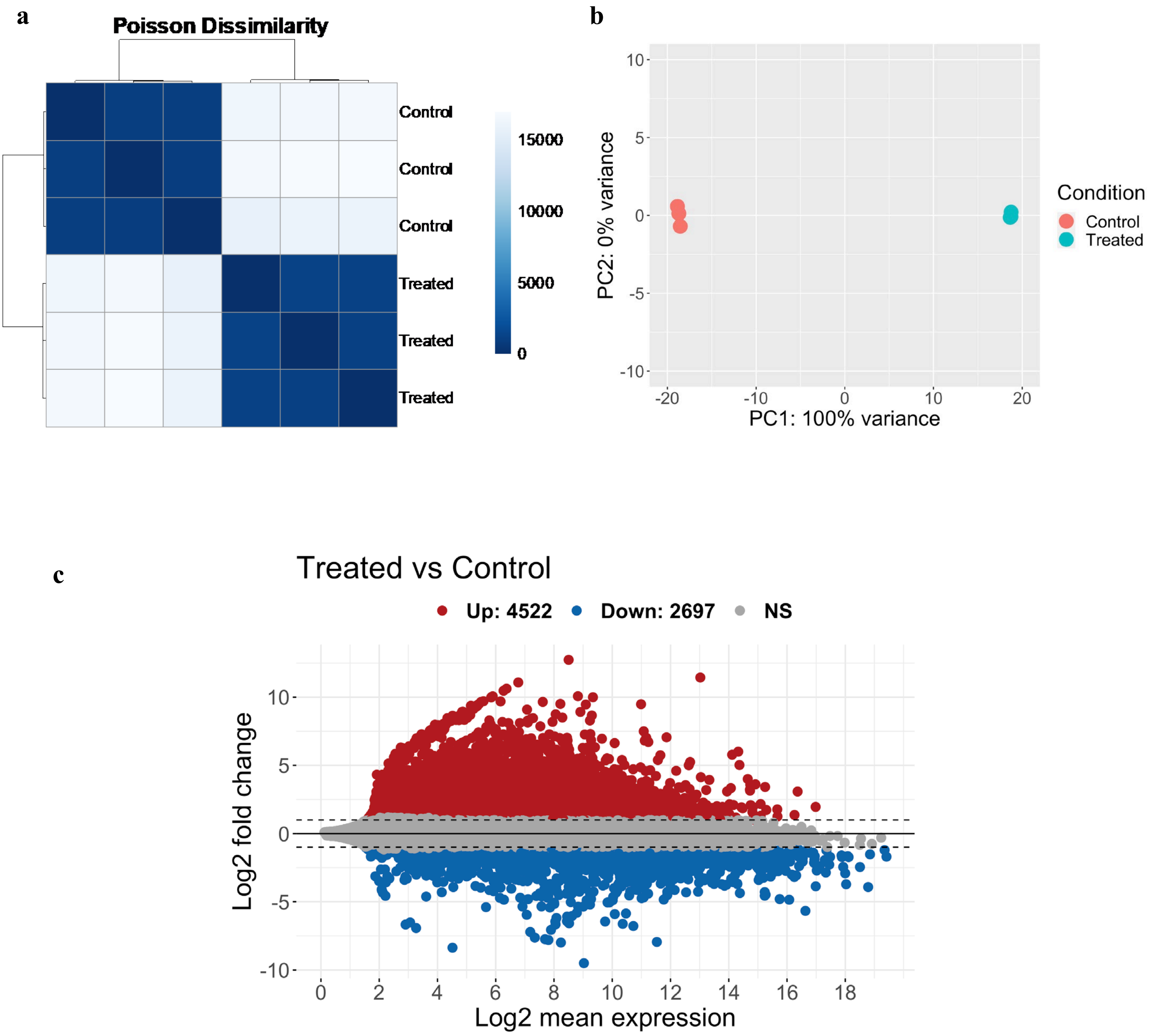
a
Poisson Dissimilarity
Control
Control
Control
Treated
Treated
Treated
15000
10000
5000
0
b
10
5
0
-5
-10
PC2: 0% variance
-20
-10
0
10
20
PC1: 100% variance
Condition
Control
Treated
c
Treated vs Control
Up: 4522
Down: 2697
NS
Log2 fold change
Log2 mean expression
0
2
4
6
8
10
12
14
16
18

GSEA: Gene Ontology Biological Process

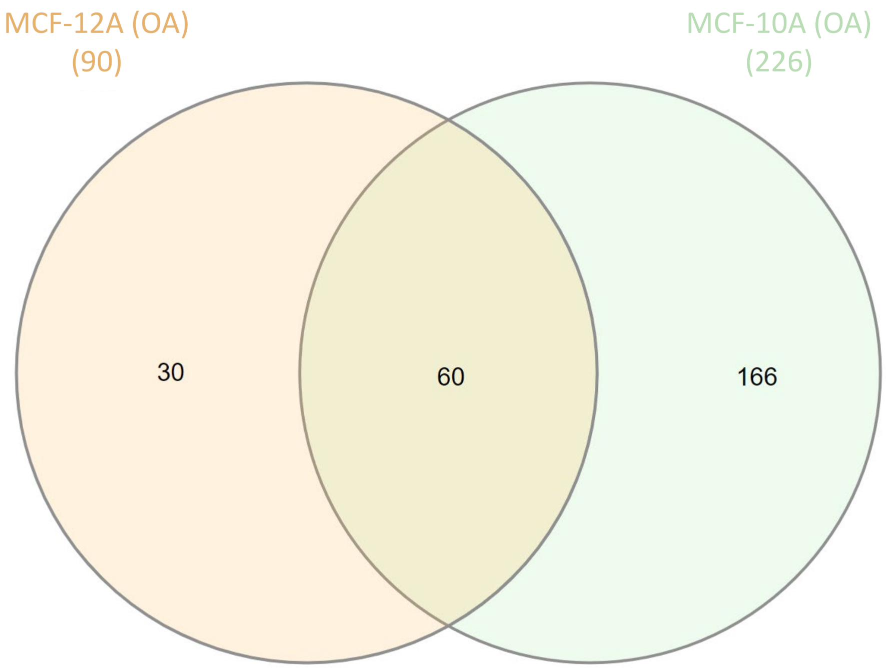

Overlapping gene sets

GOBP_TRANSMISSION_OF_NERVE_IMPULSE
GOBP_NEUROTRANSMITTER_RECEPTOR_TRANSPORT
GOBP_SENSORY_PERCEPTION_OF_PAIN
GOBP_INNER_EAR_MORPHOGENESIS
GOBP_REGULATION_OF_NEUROTRANSMITTER_RECEPTOR_ACTIVITY
GOBP_NEUROTRANSMITTER_TRANSPORT
GOBP_ORGANIC_CATION_TRANSPORT
GOBP_GLUTAMATE_RECEPTOR_SIGNALING_PATHWAY
GOBP_SENSORY_PERCEPTION_OF_MECHANICAL_STIMULUS
GOBP_REGULATION_OF_CATION_CHANNEL_ACTIVITY
GOBP_REGULATION_OF_NERVOUS_SYSTEM_PROCESS
GOBP_REGULATION_OF_NEUROTRANSMITTER_LEVELS
GOBP_CYCLIC_NUCLEOTIDE_METABOLIC_PROCESS
GOBP_CALCIUM_ION_REGULATED_EXOCYTOSIS
GOBP_REGULATION_OF_POSTSYNAPTIC_MEMBRANE_POTENTIAL
GOBP_SECOND_MESSENGER_MEDIATED_SIGNALING
GOBP_REGULATION_OF_TRANS_SYNAPTIC_SIGNALING
GOBP_REGULATION_OF_NEURONAL_SYNAPTIC_PLASTICITY
GOBP_POSITIVE_REGULATION_OF_SYNAPTIC_TRANSMISSION
GOBP_SENSORY_PERCEPTION
GOBP_NEUROTRANSMITTER_SECRETION
GOBP_CELL_FATE_SPECIFICATION
GOBP_LOCOMOTORY_BEHAVIOR
GOBP_REGULATION_OF_SYNAPTIC_PLASTICITY
GOBP_SYNAPSE_ASSEMBLY
GOBP_MULTICELLULAR_ORGANISMAL_SIGNALING
GOBP_REGULATION_OF_CALCIUM_ION_DEPENDENT_EXOCYTOSIS
GOBP_ADENYLATE_CYCLASE_MODULATING_G_PROTEIN_COUPLED_RECEPTOR_SIGNALING_PATHWAY
GOBP_DETECTION_OF_STIMULUS_INVOLVED_IN_SENSORY_PERCEPTION
GOBP_MEMBRANE_DEPOLARIZATION_DURING_ACTION_POTENTIAL
GOBP_MONOAMINE_TRANSPORT
GOBP_REGULATION_OF_HEART_CONTRACTION
GOBP_CALCIUM_MEDIATED_SIGNALING
GOBP_REGULATION_OF_BLOOD_CIRCULATION
GOBP_CHEMICAL_SYNAPTIC_TRANSMISSION_POSTSYNAPTIC
GOBP_INORGANIC_ION_IMPORT_ACROSS_PLASMA_MEMBRANE
GOBP_CELL_FATE_COMMITMENT
GOBP_SYNAPTIC_TRANSMISSION_GLUTAMATERGIC
GOBP_MEMBRANE_DEPOLARIZATION
GOBP_ACTION_POTENTIAL
GOBP_REGULATION_OF_AXONOGENESIS
GOBP_ADENYLATE_CYCLASE_INHIBITING_G_PROTEIN_COUPLED_RECEPTOR_SIGNALING_PATHWAY
GOBP_EXPORT_ACROSS_PLASMA_MEMBRANE
GOBP_POTASSIUM_ION_TRANSPORT
GOBP_BEHAVIOR
GOBP_POTASSIUM_ION_IMPORT_ACROSS_PLASMA_MEMBRANE
GOBP_LONG_TERM_SYNAPTIC_POTENTIATION
GOBP_NEURON_PROJECTION_GUIDANCE
GOBP_VESICLE_MEDIATED_TRANSPORT_IN_SYNAPSE
GOBP_AMINE_TRANSPORT
GOBP_SMOOTH_MUSCLE_CELL_DIFFERENTIATION
GOBP_EXCRETION
GOBP_MECHANORECEPTOR_DIFFERENTIATION
GOBP_NERVE_DEVELOPMENT
GOBP_DOPAMINE_TRANSPORT
GOBP_REGULATION_OF_SYSTEM_PROCESS
GOBP_CIRCULATORY_SYSTEM_PROCESS
GOBP_REGULATION_OF_SYNAPTIC_TRANSMISSION_GLUTAMATERGIC
GOBP_CATECHOLAMINE_SECRETION
GOBP_INNER_EAR_AUDITORY_RECEPTOR_CELL_DIFFERENTIATION

Figure S2a. Overlap between Gene Ontology Biological Process significantly (FDR < 0.05) associated with C8 exposure in MCF-10A and MCF-12A cell lines. InteractiVenn [1] was used to show the overlap.

GSEA: Reactome

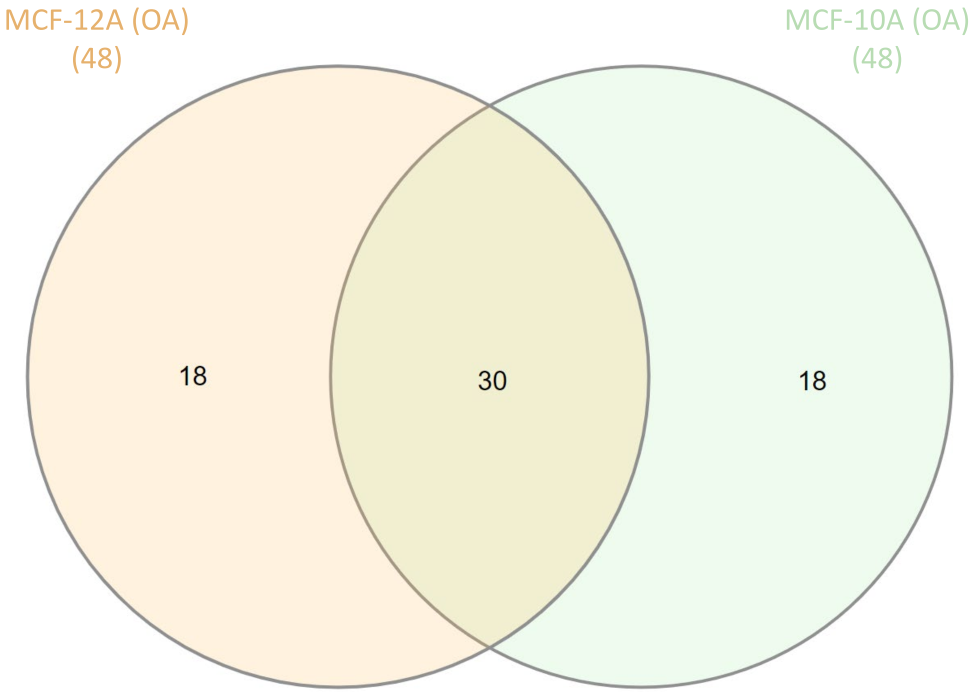

Overlapping gene sets
REACTOME_POTASSIUM_CHANNELS
REACTOME_NEURONAL_SYSTEM
REACTOME_VOLTAGE_GATED_POTASSIUM_CHANNELS
REACTOME_CARDIAC_CONDUCTION
REACTOME_NCAM_SIGNALING_FOR_NEURITE_OUT_GROWTH
REACTOME_PHASE_0_RAPID_DEPOLARISATION
REACTOME_TRANSMISSION_ACROSS_CHEMICAL_SYNAPSES
REACTOME_MUSCLE_CONTRACTION
REACTOME_CLASS_B_2_SECRETIN_FAMILY_RECEPTORS
REACTOME_REGULATION_OF_INSULIN_SECRETION
REACTOME_NCAM1_INTERACTIONS
REACTOME_NEUROTRANSMITTER_RECEPTORS_AND_POSTSYNAPTIC_SIGNAL_TRANSMISSION
REACTOME_G_ALPHA_S_SIGNALLING_EVENTS
REACTOME_GPCR_LIGAND_BINDING
REACTOME_SENSORY_PROCESSING_OF_SOUND
REACTOME_ACTIVATION_OF_MATRIX_METALLOPROTEINASES
REACTOME_PROTEIN_PROTEIN_INTERACTIONS_AT_SYNAPSES
REACTOME_INWARDLY_RECTIFYING_K_CHANNELS
REACTOME_G_ALPHA_Z_SIGNALLING_EVENTS
REACTOME_GABA_RECEPTOR_ACTIVATION
REACTOME_NEUREXINS_AND_NEUROLIGINS
REACTOME_PLATELET_HOMEOSTASIS
REACTOME_NUCLEAR_RECEPTOR_TRANSCRIPTION_PATHWAY
REACTOME_SIGNALING_BY_GPCR
REACTOME_TNFS_BIND_THEIR_PHYSIOLOGICAL_RECEPTORS
REACTOME_INTERACTION_BETWEEN_L1_AND_ANKYRINS
REACTOME_COLLAGEN_DEGRADATION
REACTOME_ADRENALINE_NORADRENALINE_INHIBITS_INSULIN_SECRETION
REACTOME_ANTIGEN_ACTIVATES_B_CELL_RECEPTOR_BCR_LEADING_TO_GENERATION_OF_SECOND_MESSENGERS
REACTOME_IMMUNOREGULATORY_INTERACTIONS_BETWEEN_A_LYMPHOID_AND_A_NON_LYMPHOID_CELL

Figure S2b. Overlap between Reactome gene sets significantly (FDR < 0.05) associated with C8 exposure in MCF-10A and MCF-12A cell lines. InteractiVenn [1] was used to show the overlap.

GSEA: KEGG pathways

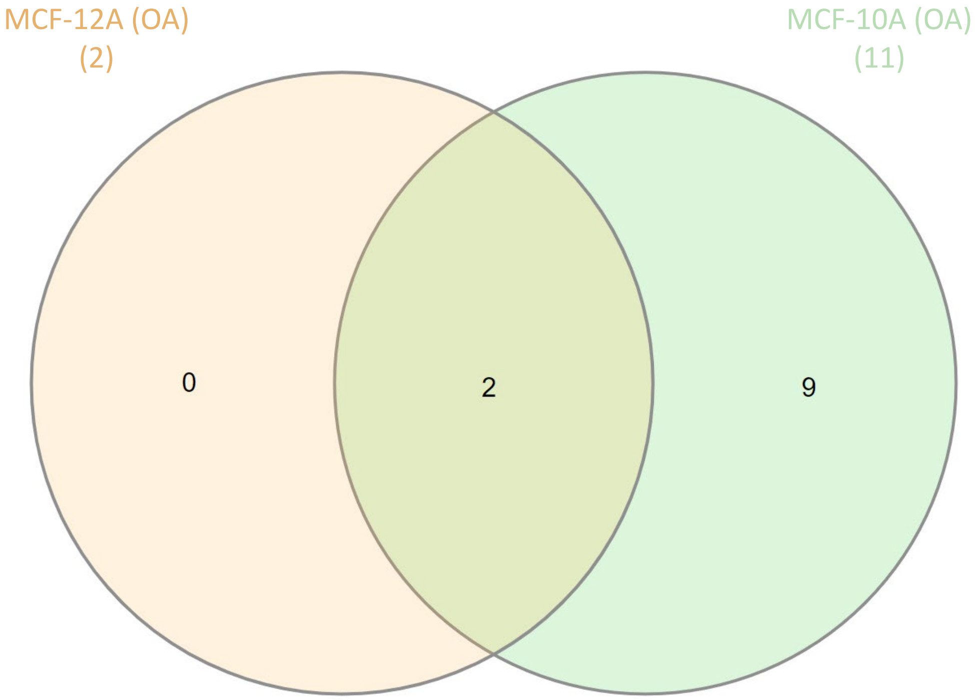

Overlapping gene sets
KEGG_NEUROACTIVE_LIGAND_RECEPTOR_INTERACTION
KEGG_CALCIUM_SIGNALING_PATHWAY

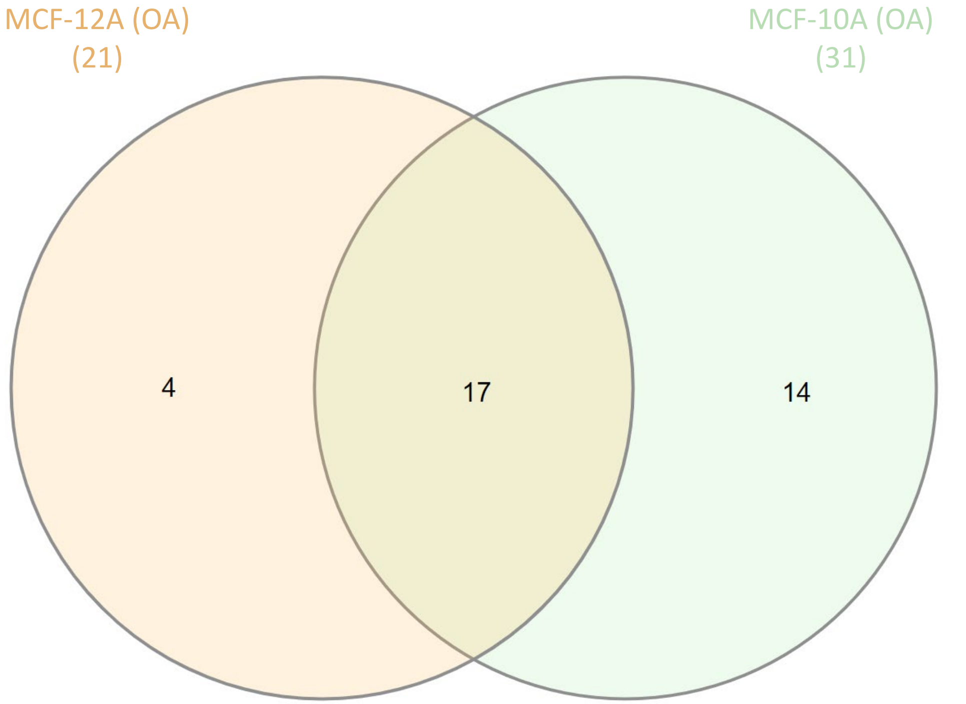

Overlapping gene sets
KEGG_NEUROACTIVE_LIGAND_RECEPTOR_INTERACTION
KEGG_CALCIUM_SIGNALING_PATHWAY
KEGG_LONG_TERM_DEPRESSION
KEGG_BASAL_CELL_CARCINOMA
KEGG_CYTOKINE_CYTOKINE_RECEPTOR_INTERACTION
KEGG_PPAR_SIGNALING_PATHWAY
KEGG_CELL_ADHESION_MOLECULES_CAMS
KEGG_B_CELL_RECEPTOR_SIGNALING_PATHWAY
KEGG_AXON_GUIDANCE
KEGG_MELANOGENESIS
KEGG_HEDGEHOG_SIGNALING_PATHWAY
KEGG_NATURAL_KILLER_CELL_MEDIATED_CYTOTOXICITY
KEGG_ARRHYTHMOGENIC_RIGHT_VENTRICULAR_CARDIOMYOPATHY_ARVC
KEGG_DORSO_VENTRAL_AXIS_FORMATION
KEGG_VEGF_SIGNALING_PATHWAY
KEGG_MAPK_SIGNALING_PATHWAY
KEGG_LONG_TERM_POTENTIATION

Figure S2c. Overlap between KEGG pathways significantly associated with C8 exposure in MCF-10A and MCF-12A cell lines. FDR < 0.05 (LEFT) and FDR < 0.25 (RIGHT). InteractiVenn [1] was used to show the overlap.

REFERENCES TO SUPPLEMENTARY FIGURE 2

1. Heberle, H.; Meirelles, G. V.; da Silva, F. R.; Telles, G. P.; Minghim, R. ***InteractiVenn: a web-based tool for the analysis of sets through Venn diagrams***. BMC Bioinformatics 16:169 (2015)

a

	N	Overall Pop: (n=84)	ER+ Pop: (n=28)	ER- Pop: (n=28)	Control Pop: (n=28)
Age					
	Median	50	51	50	50
	Range	(34-65)	(38-65)	(34-63)	(36-63)
BMI					
	Median	26	26	23	29
	Range	(17-41)	(18-40)	(17-41)	(21-41)
ER (%)					
	0	28 (33.33)	0	28 (100)	0
	1	28 (33.33)	28 (100)	0	0
	n/a	28 (33.33)	0	0	28 (100)
MenopauseStatus (%)					
	post	36 (42.86)	10 (35.71)	15 (53.57)	11 (39.29)
	post (hysterectomy)	13 (15.48)	7 (25)	1 (3.57)	5 (17.86)
	post (medication)	5 (5.95)	1 (3.57)	2 (7.14)	2 (7.14)
	pre	1 (1.19)	0	1 (3.57)	0
	pre(1)	7 (8.33)	1 (3.57)	1 (3.57)	5 (17.86)
	pre(2)	11 (13.1)	4 (14.29)	5 (17.86)	2 (7.14)
	pre(3)	11 (13.1)	5 (17.86)	3 (10.71)	3 (10.71)
MensmenoCode (%)					
	Early_Follicular	7 (8.33)	1 (3.57)	1 (3.57)	5 (17.86)
	Late_Follicular	11 (13.1)	4 (14.29)	5 (17.86)	2 (7.14)
	Luteal	11 (13.1)	5 (17.86)	3 (10.71)	3 (10.71)
	NA	1 (1.19)	0	1 (3.57)	0
	Post_menopausal	54 (64.29)	18 (64.29)	18 (64.29)	18 (64.29)
obcode (%)					
	NA	4 (4.76)	0	2 (7.14)	2 (7.14)
	NW	32 (38.1)	12 (42.86)	15 (53.57)	5 (17.86)
	OB	27 (32.14)	8 (28.57)	7 (25)	12 (42.86)
	OW	21 (25)	8 (28.57)	4 (14.29)	9 (32.14)
Race (%)					
	AA	13 (15.48)	3 (10.71)	3 (10.71)	7 (25)
	Cauc	70 (83.33)	24 (85.71)	25 (89.29)	21 (75)
	Other	1 (1.19)	1 (3.57)	0	0

b

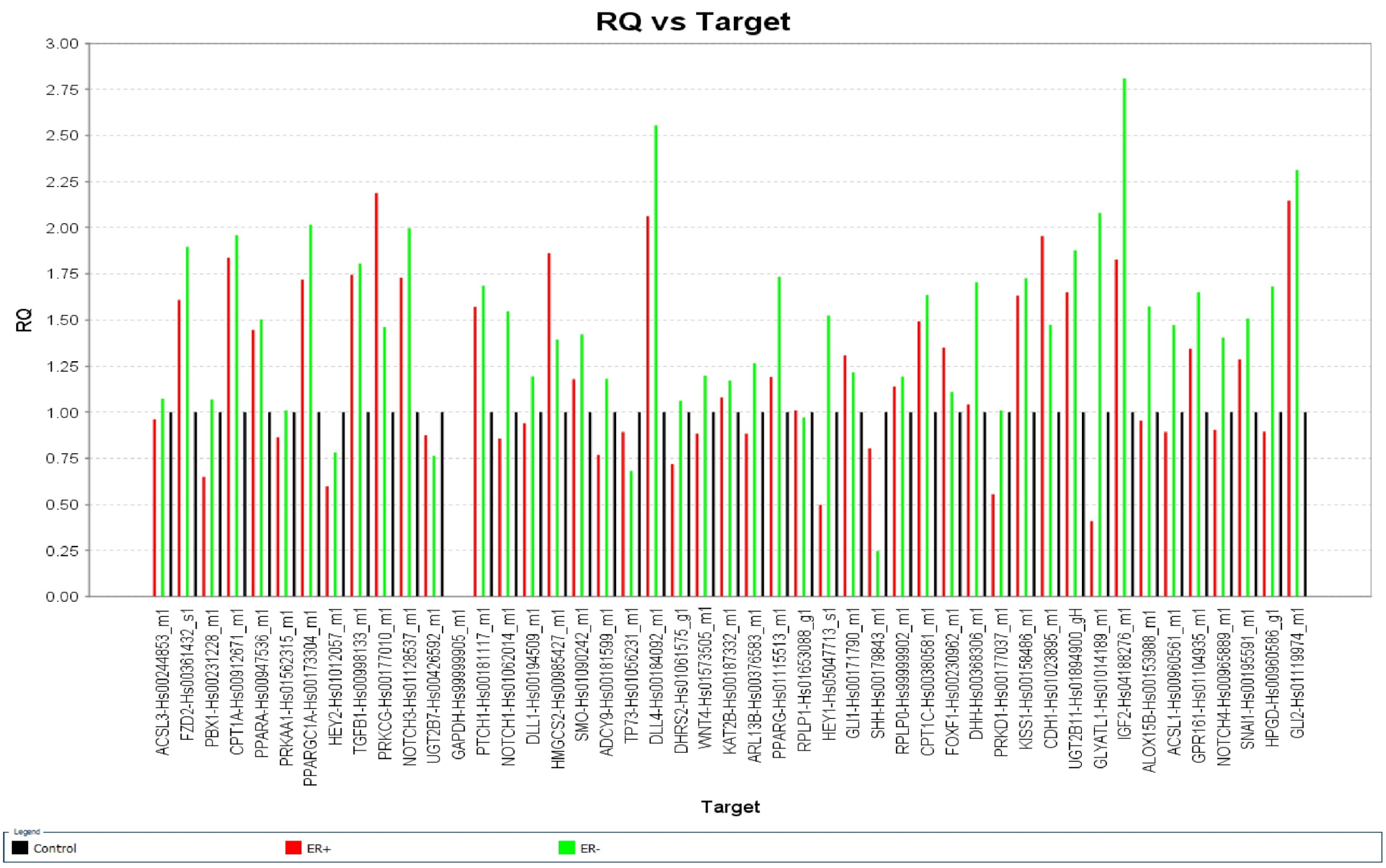

a

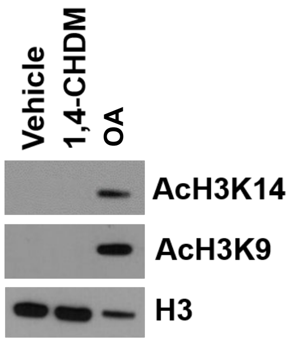

b

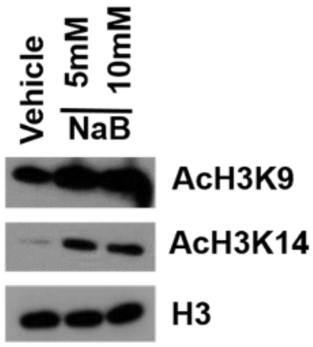

c

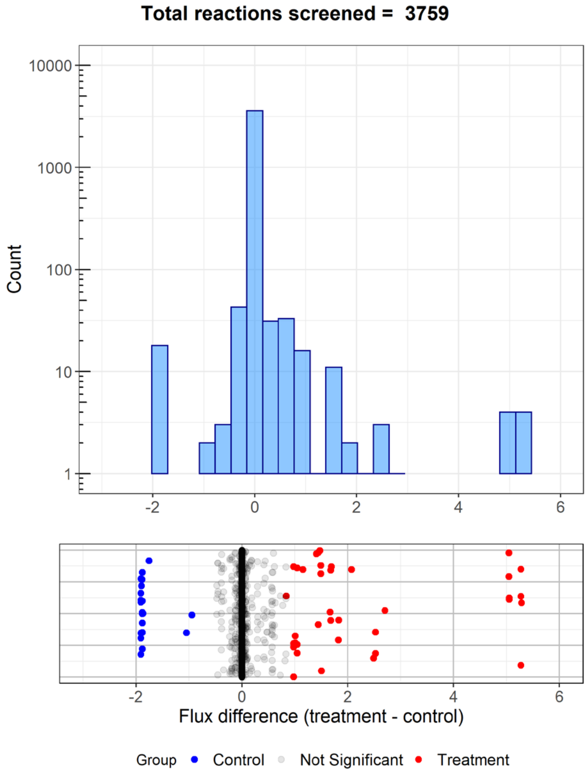

Subsystem	Reaction example	n
Triacylglycerol Synthesis	Diacylglycerol acyltransferase	7
Vitamin A Metabolism	Retinyl ester hydrolase	5
Fatty Acid Metabolism	Electron transfer flavoprotein	4
Pyruvate Metabolism	D-lactate dehydrogenase	4
Methionine Metabolism	Adenosylhomocysteinase	3
Folate Metabolism	Methenyltetrahydrofolate cyclohydrolase, mitochondrial	2
Glycerophospholipid Metabolism	Glycerol kinase	2
Miscellaneous	Peroxidase (multiple substrates)	2
Oxidative Phosphorylation	Ubiquinol-6 cytochrome c reductase, Complex III	2
Glycine, Serine, and Threonine Metabolism	Glycine N-methyltransferase	1
Glycolysis/ Gluconeogenesis	Glycerol-3-phosphate dehydrogenase (FAD), mitochondrial	1
Inositol Phosphate Metabolism	Inositol oxygenase	1
Methylation	Histone methylation	1
ROS Detoxification	Catalase	1
Transport, Endoplasmic Reticular	S-Adenosyl-L-methionine intracellular diffusion	1
Urea cycle/amino group metabolism	Sarcosine dehydrogenase (m)	1

Subsystem	Reaction example	n
Nucleotides	Nucleoside-diphosphatase (dUDP)	12
Glycolysis/ Gluconeogenesis	Glyceraldehyde-3-phosphate dehydrogenase	5
Pentose Phosphate Pathway	Deoxyribokinase	2
Oxidative Phosphorylation	NADH dehydrogenase, mitochondrial	1

d

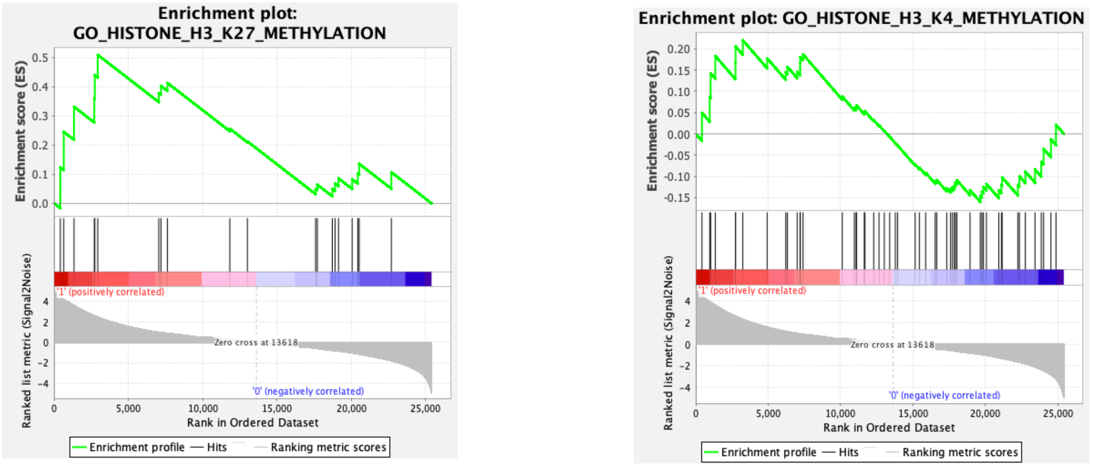

e

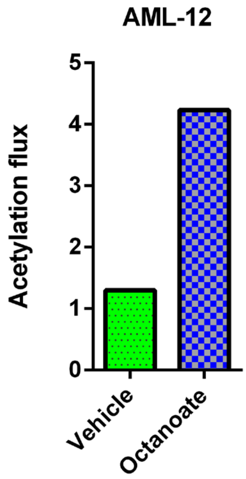

Supplementary File 1

Gene Symbol	Assay ID
HMGCS2	Hs00985427_m1
ACSL3	Hs00244853_m1
CPT1B	Hs03046298_s1
DLL4	Hs00184092_m1
HEY1	Hs05047713_s1
HEY 2	Hs01012057_m1
Notch 1	Hs01062014_m1
RPLP1	Hs01653088_g1

Supplementary File 2

Gene Symbol	Assay ID
HMGCS2	Hs00985427_m1
DHRS2	Hs01061575_g1
HPGD	Hs00960586_g1
UGT2B11	Hs01894900_gH
ALOX15B	Hs00153988_m1
UGT2B7	Hs00426592_m1
GLYATL1	Hs01014189_m1
PBX1	Hs00231228_m1
ACSL3	Hs00244853_m1
ACSL1	Hs00960561_m1
CPT1A	Hs00912671_m1
CPT1C	Hs00380581_m1
GPR161	Hs01104935_m1
KISS1	Hs00158486_m1
SHH	Hs00179843_m1
DHH	Hs00368306_m1
PTCH1	Hs00181117_m1
SMO	Hs01090242_m1
GLI1	Hs00171790_m1
GLI2	Hs01119974_m1
FOXF1	Hs00230962_m1
FZD2	Hs00361432_s1
p73	Hs01056231_m1
DLL1	Hs00194509_m1
DLL4	Hs00184092_m1
PCAF	Hs00187332_m1
HEY1	Hs05047713_s1
HEY 2	Hs01012057_m1
NOTCH4	Hs00965889_m1
NOTCH3	Hs01128537_m1
NOTCH1	Hs01062014_m1
TGFB1	Hs00998133_m1
SNAI1	Hs00195591_m1
CDH1	Hs01023895_m1
PPARγ	Hs01115513_m1
PPARα	Hs00947536_m1
PPARGC1A	Hs00173304_m1
ADCY9	Hs00181599_m1
IGF-2	Hs04188276_m1
PRKCG	Hs00177010_m1

PRKD1	Hs00177037_m1
WNT-4	Hs01573505_m1
AMPK	Hs01562315_m1
Arl13b	Hs00376583_m1
RPLP0	Hs99999902_m1
RPLP1	Hs01653088_g1
GAPDH	Hs99999905_m1

Original Blots Figure 6A
MCF-10A

AcH3K9

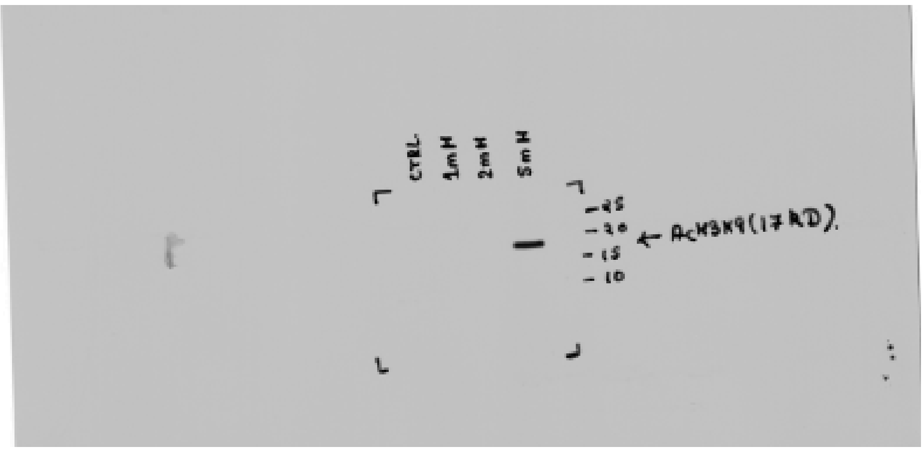

AcH3K14

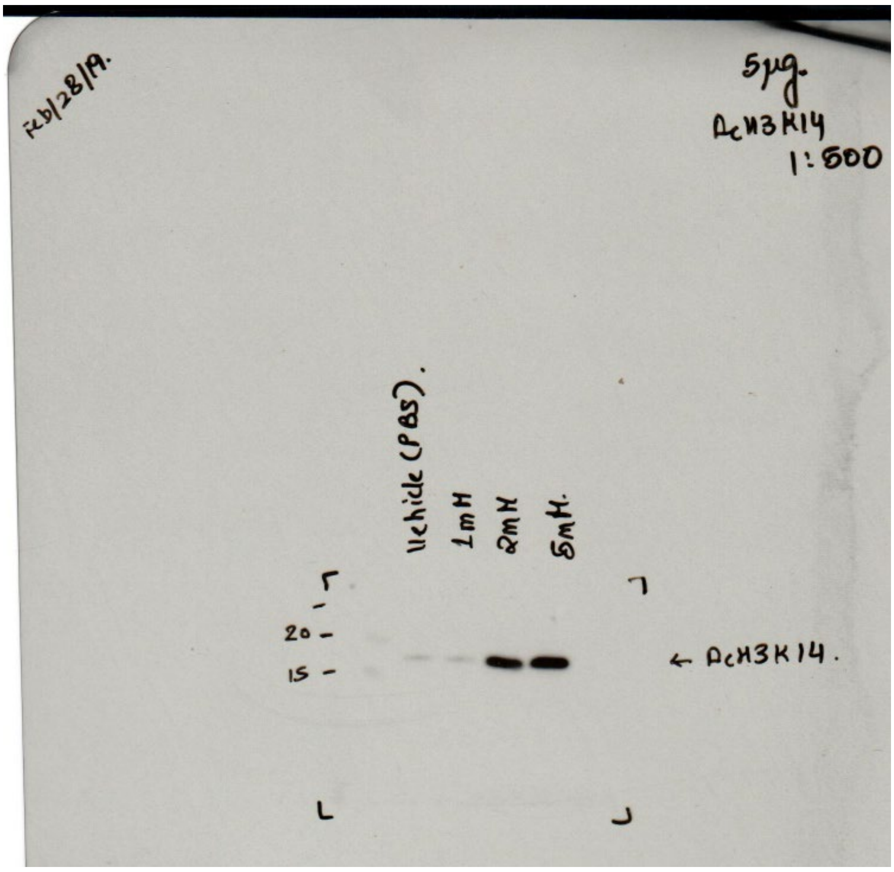

H3

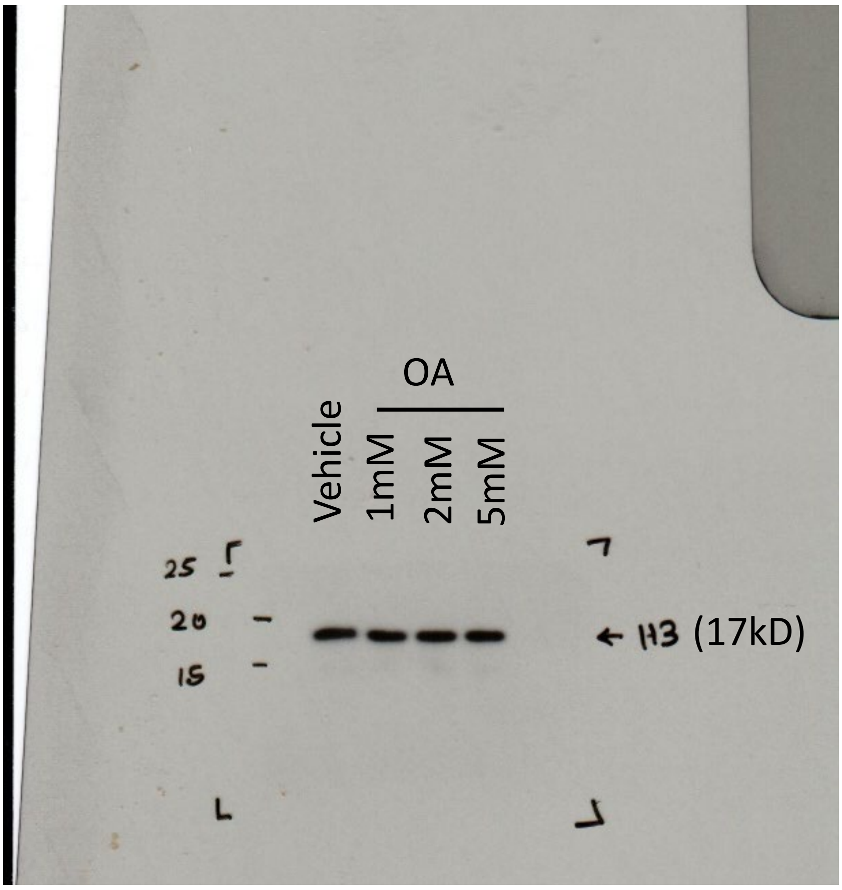

Original Blots Figure 6A
MCF-10A

AcH3K9/ AcH3K14

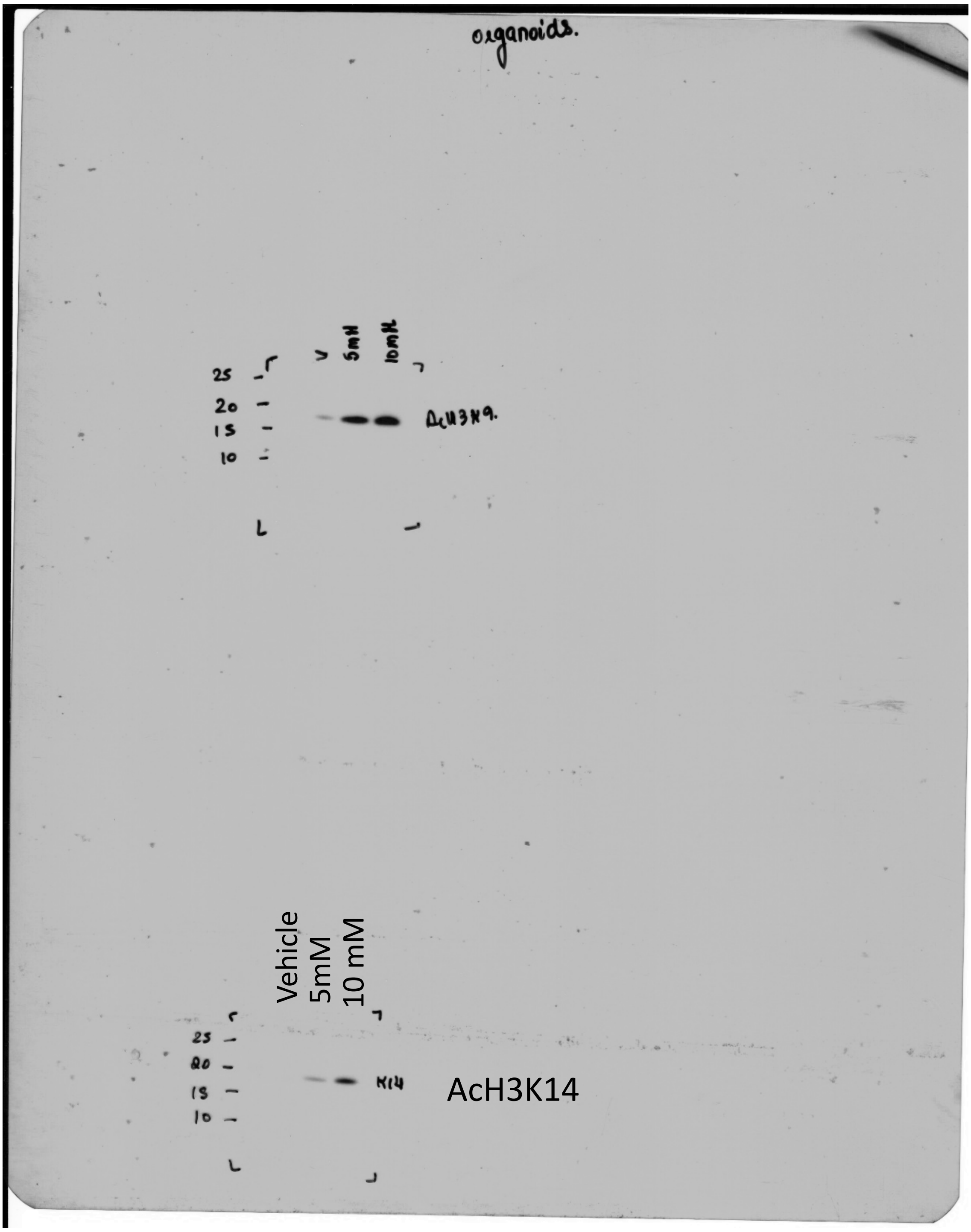

H3

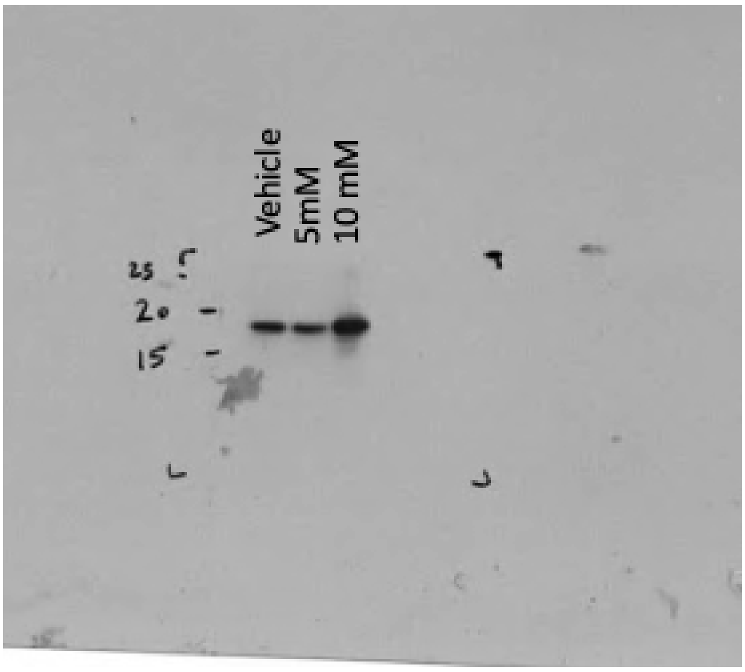

Original Blots Figure 6B
LA in 10A and organoids

AcH3K14

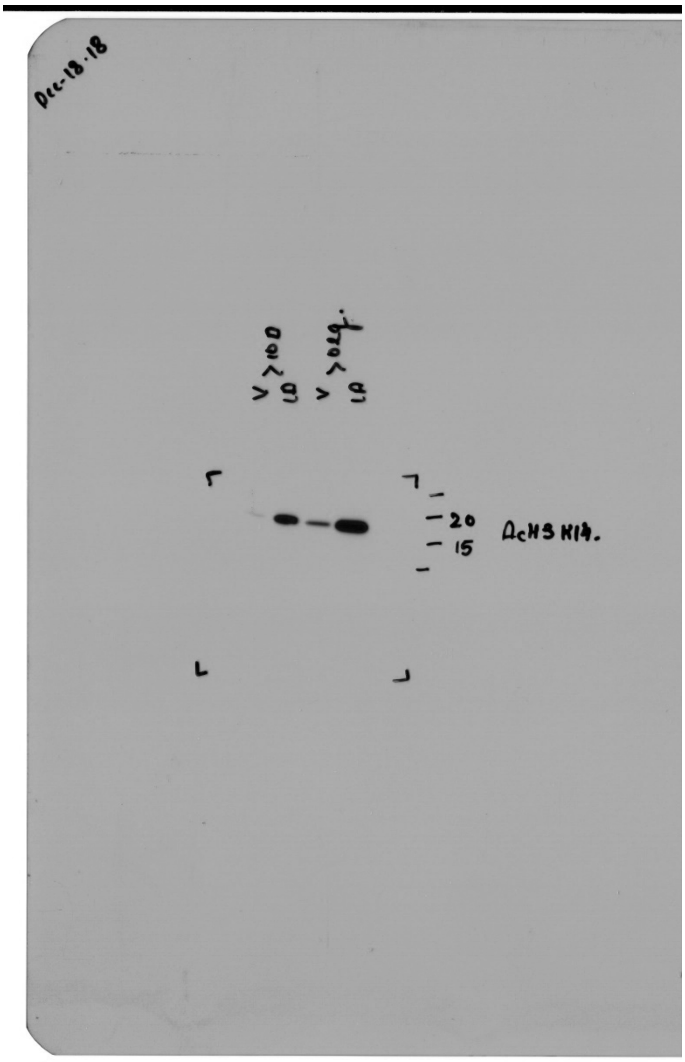

AcH3K9

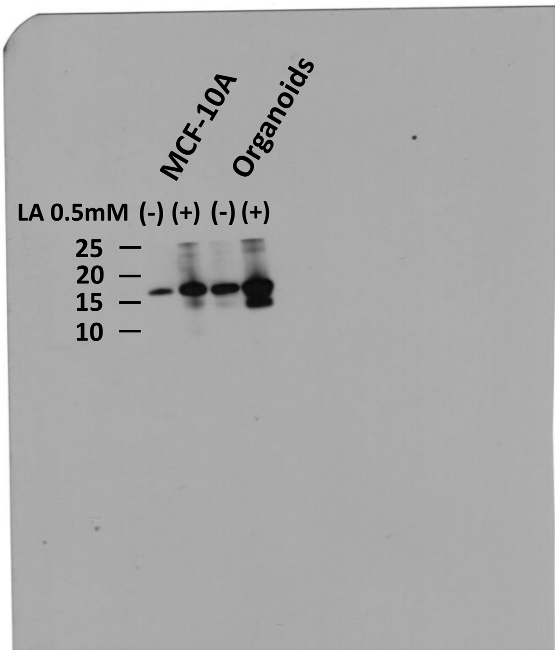

H3

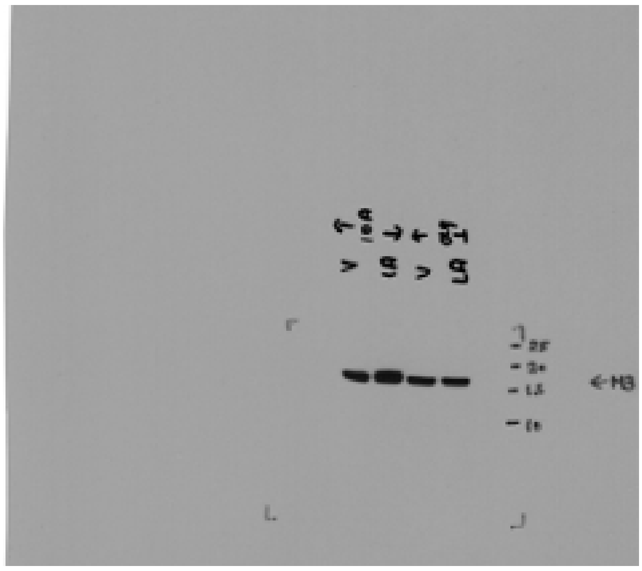

Supplement: Supplementary file 1 — Supplementary Figures and Files [file 41523_2022_422_MOESM1_ESM.pdf]
