# Supplementary material for: Lipid exposure activates gene expression changes associated with estrogen receptor negative breast cancer
Source: NPJ Breast Cancer. 2022 May 4;8:59. doi: 10.1038/s41523-022-00422-0 (PMC9068822; doi:10.1038/s41523-022-00422-0)
Supplement: Supplementary file 2 — Reporting Summary [file 41523_2022_422_MOESM2_ESM.pdf]

nature research

Corresponding author(s): Susan E. Clare, Seema A. Khan

Last updated by author(s): Dec 22, 2020

Reporting Summary

Nature Research wishes to improve the reproducibility of the work that we publish. This form provides structure for consistency and transparency in reporting. For further information on Nature Research policies, see our Editorial Policies and the Editorial Policy Checklist.

Statistics

For all statistical analyses, confirm that the following items are present in the figure legend, table legend, main text, or Methods section.

n/a	Confirmed	
☐	☒	The exact sample size (n) for each experimental group/condition, given as a discrete number and unit of measurement
☐	☒	A statement on whether measurements were taken from distinct samples or whether the same sample was measured repeatedly
☐	☒	The statistical test(s) used AND whether they are one- or two-sided<br>*Only common tests should be described solely by name; describe more complex techniques in the Methods section.*
☐	☒	A description of all covariates tested
☐	☒	A description of any assumptions or corrections, such as tests of normality and adjustment for multiple comparisons
☐	☒	A full description of the statistical parameters including central tendency (e.g. means) or other basic estimates (e.g. regression coefficient) AND variation (e.g. standard deviation) or associated estimates of uncertainty (e.g. confidence intervals)
☐	☒	For null hypothesis testing, the test statistic (e.g. F, t, r) with confidence intervals, effect sizes, degrees of freedom and P value noted<br>*Give P values as exact values whenever suitable.*
☒	☐	For Bayesian analysis, information on the choice of priors and Markov chain Monte Carlo settings
☒	☐	For hierarchical and complex designs, identification of the appropriate level for tests and full reporting of outcomes
☒	☐	Estimates of effect sizes (e.g. Cohen's d, Pearson's r), indicating how they were calculated

Our web collection on statistics for biologists contains articles on many of the points above.

Software and code

Policy information about availability of computer code

Data collection	The Methods section provides a list of software and corresponding versions utilized.
Data analysis	The Methods section gives a complete list of software packages capable of reproducing the analyses and figures.

For manuscripts utilizing custom algorithms or software that are central to the research but not yet described in published literature, software must be made available to editors and reviewers. We strongly encourage code deposition in a community repository (e.g. GitHub). See the Nature Research guidelines for submitting code & software for further information.

Data

Policy information about availability of data

All manuscripts must include a data availability statement. This statement should provide the following information, where applicable:

- Accession codes, unique identifiers, or web links for publicly available datasets
- A list of figures that have associated raw data
- A description of any restrictions on data availability

Data and materials availability: The datasets generated and analyzed during the current study are publicly available in the Gene Expression Omnibus: accession number GSE126799 (RNA-seq) and XXX (ATAC-seq).

Field-specific reporting

Please select the one below that is the best fit for your research. If you are not sure, read the appropriate sections before making your selection.

☒ Life sciences ☐ Behavioural & social sciences ☐ Ecological, evolutionary & environmental sciences

For a reference copy of the document with all sections, see nature.com/documents/nr-reporting-summary-flat.pdf

Life sciences study design

All studies must disclose on these points even when the disclosure is negative.

Sample size	RNA from tissue samples obtained 56 bilateral mastectomy cases (28 ER+ and 28 ER–) and 28 healthy RM controls were used in this study. This RNA was remaining from our previous study [PMID: 28263391] in which a number of the LiMe genes were shown to have significant differential expression when comparing ER- to ER+ disease.
Data exclusions	No data were excluded
Replication	This was a circular process in which findings in vitro were then tested in the CUB specimens and, subsequently, the findings in the CUB specimens tested in the MCF10As and organoids.
Randomization	Randomization was not relevant to this study
Blinding	Randomization was not relevant to this study

Reporting for specific materials, systems and methods

We require information from authors about some types of materials, experimental systems and methods used in many studies. Here, indicate whether each material, system or method listed is relevant to your study. If you are not sure if a list item applies to your research, read the appropriate section before selecting a response.

Materials & experimental systems

n/a	Involved in the study	
☐	☒	Antibodies
☐	☒	Eukaryotic cell lines
☒	☐	Palaeontology and archaeology
☒	☐	Animals and other organisms
☐	☒	Human research participants
☒	☐	Clinical data
☒	☐	Dual use research of concern

Methods

n/a	Involved in the study	
☒	☐	ChIP-seq
☒	☐	Flow cytometry
☒	☐	MRI-based neuroimaging

Antibodies

Antibodies used	AcH3K9 (rabbit mAb Cell Signaling #9649), AcH3K14 (rabbit mAb Cell Signaling #7627) and H3 (Rabbit mAb Cell Signaling #9715)
Validation	Manufacturer validated AcH3K9 for Western Blot using trichostatin A (TSA) to induce histone acetylation in HeLa and NIH 3T3 cells. Manufacturer validated AcH3K14 for Western Blotting using trichostatin A (TSA) to induce histone acetylation in HeLa, C2C12, and COS-7 cells. Manufacturer validated H3 for Western Blotting in K562, DAE, CTLL-2, BAEC and C6 cells. We utilized sodium butyrate to block histone deactylation to validate the AcH3K9 and AcH3K14 antibodies (Supplemental Figure 3, B.)

Eukaryotic cell lines

Policy information about cell lines

Cell line source(s)	MCF10A cell line was obtained from American Type Culture Collection (ATCC)
Authentication	cell line was not authenticated
Mycoplasma contamination	cell line was not tested for mycoplasma contamination
Commonly misidentified lines (See ICLAC register)	none

Human research participants

Policy information about studies involving human research participants

Population characteristics	Characteristics of the human research participants are provided in the table in Supplemental Figure 2 A.
Recruitment	Patients diagnosed with unilateral breast cancer and who had chosen to undergo contralateral prophylactic mastectomy were recruited at the Prentice Women's Hospital of Northwestern Medicine under an approved protocol (NU11B04), with exclusions for neoadjuvant treatment, prior endocrine therapy or pregnancy/lactation during the prior 2 years. A group of reduction mammoplasty (RM) patients were also recruited as standard risk controls.
Ethics oversight	Northwestern University

Note that full information on the approval of the study protocol must also be provided in the manuscript.
